# Single-Cell Phosphospecific Flow Cytometric Analysis of Canine and Murine Adipose-Derived Stem Cells

**DOI:** 10.1155/2017/5701016

**Published:** 2017-08-16

**Authors:** Harumichi Itoh, Shimpei Nishikawa, Tomoya Haraguchi, Yu Arikawa, Masato Hiyama, Toshie Iseri, Yoshiki Itoh, Munekazu Nakaichi, Yasuho Taura, Kenji Tani, Kazuhito Itamoto

**Affiliations:** ^1^Department of Small Animal Clinical Science, Joint Faculty of Veterinary Medicine, Yamaguchi University, 1677-1 Yoshida, Yamaguchi City, Yamaguchi 753-8515, Japan; ^2^Department of Veterinary Surgery, Joint Faculty of Veterinary Medicine, Yamaguchi University, 1677-1 Yoshida, Yamaguchi City, Yamaguchi 753-8515, Japan; ^3^Department of Veterinary Radiology, Joint Faculty of Veterinary Medicine, Yamaguchi University, 1677-1 Yoshida, Yamaguchi City, Yamaguchi 753-8515, Japan

## Abstract

This study aimed to demonstrate single-cell phosphospecific flow cytometric analysis of canine and murine adipose-derived stem/stromal cells (ADSCs). ADSCs were obtained from clinically healthy laboratory beagles and C57BL/6 mice. Cell differentiation into adipocytes, osteocytes, and chondrocytes was observed for the cultured canine ADSCs (cADSCs) and murine ADSCs (mADSCs) to determine their multipotency. We also performed single-cell phosphospecific flow cytometric analysis related to cell differentiation and stemness. Cultured cADSCs and mADSCs exhibited the potential to differentiate into adipocytes, osteocytes, and chondrocytes. In addition, single-cell phosphospecific flow cytometric analysis revealed similar *β*-catenin and Akt phosphorylation between mADSCs and cADSCs. On the other hand, it showed the phosphorylation of different Stat proteins. It was determined that cADSCs and mADSCs show the potential to differentiate into adipocytes, osteocytes, and chondrocytes. Furthermore, a difference in protein phosphorylation between undifferentiated cADSCs and mADSCs was identified.

## 1. Introduction

Adipose-derived stem/stromal cells (ADSCs) reside in adipose tissues and are considered to be the progenitors of adipocytes. A recent study revealed that ADSCs can differentiate into adipocytes, osteocytes, and chondrocytes [1]. Mesenchymal stem cells (MSCs), such as ADSCs and bone marrow-derived stem/stromal cells (BMSCs), have many clinical advantages over embryonic stem cells (ESCs) and induced pluripotent stem cells (iPSCs). As ESCs are pluripotent stem cells derived from blastocysts, the ethical issues regarding their use are still unresolved. In contrast, iPSCs are generated by transduction of the four Yamanaka factors into differentiated somatic cells [2]. However, it was suggested that they are associated with the risk of malignant transformation [3]. MSCs, on the other hand, are easily obtained from autologous tissues, thus minimizing these problems associated with ESCs and iPSCs. Additionally, ADSCs can be obtained less invasively than BMSCs as they are present in subcutaneous tissues [4]. Considering this background, ADSCs are expected to be useful in human and veterinary regenerative medicine.

The expression of cluster of differentiation (CD) molecules in murine and human ADSC has been reported. Specifically, some surface antigen markers, such as CD34, CD44, and CD90, have been detected as ADSC markers [5]. However, how these surface antigen markers are functionally related to cell differentiation or maintenance of ADSC stemness is not well understood. In contrast, the intracellular protein phosphorylation directly reflects cell differentiation, proliferation, migration, adhesion, and so on. In murine ADSCs (mADSCs), some studies have reported on protein phosphorylation as a means of differentiating adipocytes, osteocytes, chondrocytes, and others. However, most of these reports describe comparisons between before and after the induction of differentiation. Furthermore, there has been no comprehensive study on protein phosphorylation with regard to ADSCs. In veterinary medicine, multipotency to differentiate into adipocytes, osteocytes, and chondrocytes has been identified in cADSCs. However, the details of differentiation mechanism have not been well identified.

In this study, we performed a comprehensive analysis of protein phosphorylation in mADSCs and cADSCs. We also compared protein phosphorylation between these two types of cell.

## 2. Materials and Methods

### 2.1. Ethical Approval

The institutional animal experiment ethics committee approved the animal experiments in this study. This work was conducted in accordance with the institutional guidelines of Yamaguchi University.

### 2.2. Animals and ADSC Isolation

Subcutaneous adipose tissue was collected from clinically healthy laboratory beagles (an 8-year-old female weighing 9.3 kg and a 6-year-old male weighing 9.0 kg) and C57BL/6J mice purchased from a private company (Kyudo Co., Ltd., Saga, Japan). cADSCs and mADSCs were isolated as previously described [6]. To obtain adipose tissues from laboratory beagles, anesthesia was induced in the dogs using 7 mg/kg propofol (1% propofol injection; Intervet, Tokyo, Japan) and maintained with isoflurane (Isoflurane, DS Pharma Animal Health Co., Osaka, Japan) in oxygen. In addition, 20 *μ*g/kg buprenorphine (Lepetan® Otsuka Pharmaceutical, Tokyo, Japan) and 0.2 mg/kg meloxicam (Metacam® Boehringer Ingelheim, Tokyo, Japan) were used as analgesics. Adipose tissue was washed with Dulbecco's phosphate-buffered saline (DPBS; Wako, Osaka, Japan) and cut into fine pieces that were incubated at 37.5°C for 1 h with shaking in high-glucose Dulbecco's modified Eagle's medium (Wako) supplemented with 10% fetal bovine serum (FBS; Sigma-Aldrich, St. Louis, MO, USA), penicillin (100 U/mL)/streptomycin (100 *μ*g/mL), amphotericin B (0.25 *μ*g/mL) (100x Antibiotic-Antimycotic Mixed Stock Solution; Nacalai Tesque, Kyoto, Japan), and collagenase type I (1.0 mg/mL; Sigma-Aldrich). A 100-*μ*m nylon mesh (EASYstrainer, 100 *μ*m; Greiner Bio-One Japan, Tokyo, Japan) was used to filter digested tissues, followed by centrifugation at 1,800 rpm for 5 min in FACS buffer composed of 30 mL of DPBS supplemented with 1% FBS and 1 mM EDTA·3Na (Wako). The pellet was seeded on culture plates and reached 80%–90% confluence; ADSCs were replated using trypsin-EDTA (0.25%).

### 2.3. Cell Differentiation and Immunofluorescence Staining

A cell differentiation kit (Mouse Mesenchymal Stem Cell Functional Identification Kit; R&D Systems, Minneapolis, MN, USA) was used to analyze the adipogenic, osteogenic, and chondrogenic differentiation potential of ADSCs, in accordance with the manufacturer's instructions.

To detect adipogenic differentiation by immunocytochemistry, cells were incubated for 1 h in DPBS containing 10 *μ*g/mL goat anti-mouse fatty acid-binding protein (FABP) 4 antibody to label adipocytes. Cells were washed with DPBS and incubated for 1 h in DPBS containing phycoerythrin- (PE-) conjugated secondary antibody [rabbit F(ab′)2 anti-goat IgG H&L (PE), preadsorbed; Abcam® Japan, Tokyo, Japan]. After washing with DPBS, cells were mounted with a solution containing 5 *μ*g/mL Hoechst 33342 (Dojindo Laboratories, Kumamoto, Japan) to stain nuclei.

To detect osteogenic differentiation by immunocytochemistry, cells were then incubated for 1 h in DPBS containing 10 *μ*g/mL goat anti-mouse osteopontin antibody to label osteocytes. After washing with DPBS, cells were incubated for 1 h in DPBS containing PE-conjugated rabbit anti-goat secondary antibody, washed with DPBS, and mounted as described above.

To detect chondrogenic differentiation by immunocytochemistry, the pellet was fixed and cut into sections with a thickness of 5–10 *μ*m using a specimen matrix [Embedding Medium for Frozen Tissue Specimens to ensure Optimal Cutting Temperature (OCT); Sakura Finetek USA, Torrance, CA, USA] and a cryostat (CM 1950; Leica, Melbourne, Australia). After cutting into sections and placing on a slide, the slide was incubated for 1 h in DPBS containing 10 *μ*g/mL sheep anti-mouse collagen II antibody to label chondrocytes. After washing with DPBS, cells were incubated for 1 h in DPBS containing PE-conjugated donkey anti-sheep secondary antibody [Donkey F(ab′)2 Anti-Sheep IgG H&L (Phycoerythrin) preadsorbed; Abcam], washed with DPBS, and mounted as described above. For a negative control, we used DPBS without a primary antibody for all differentiation potentials.

### 2.4. Single-Cell Phosphospecific Flow Cytometric Analysis

Adherent cells from passage 4 were dissociated and cells were resuspended and incubated in FACS buffer containing 2 *μ*L of Fc receptor-blocking reagent (FcX Blocker; Biolegend, San Diego, CA, USA). Cells were stained with reagent to exclude dead cells (Zombie NIR; Tomy Digital Biology Co., Ltd., Tokyo, Japan). After washing the cells, IC fixation buffer (Affymetrix Japan, Tokyo, Japan) was added with shaking and then incubated. Permeabilization buffer (Affymetrix Japan) was diluted with purified water 10 times and added to wash the cells. After permeabilization, antibodies were used to stain each phosphorylated protein, as shown in Table 1. All antibodies were supplied by BD Biosciences (Tokyo, Japan). Stained cells were sorted by flow cytometry (Accuri C6; BD) and data were analyzed with FlowJo software (Tree Star, Ashland, OR, USA). Phosphorylation of proteins was considered to be positive when over 10% of all cells were stained, in contrast to the isotype control.

## 3. Results

### 3.1. Cell Differentiation into Adipocytes, Osteocytes, and Chondrocytes

**Figure 1:**
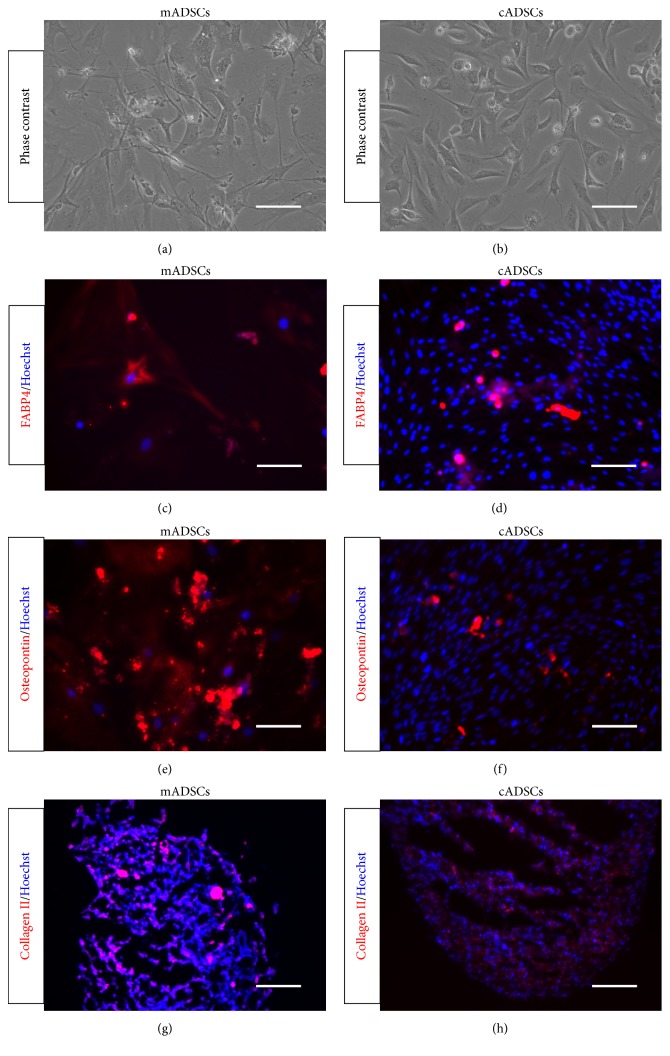
Differentiation potential of mADSCs (a, c, e, g) and cADSCs (b, d, f, h). Spindle-shaped morphology was identified by phase-contrast microscopy (a, b). FABP4 (c, d), osteopontin (e, f), and collagen II expression (red) following adipogenic (c, d), osteogenic (e, f), and chondrogenic (g, h) differentiation for mADSCs and cADSCs, as determined by immunocytochemistry. The nuclei were stained with Hoechst 33342 (blue).

Both mADSCs and cADSCs passaged four times and displayed spindle-shaped morphology, as determined with phase-contrast microscopy (Figures 1(a) and 1(b)). To assess the adipogenic, osteogenic, and chondrogenic differentiation potentials of cells, cADSCs and mADSCs were differentiated into these lineages using in vitro differentiation to each subpopulation. After this in vitro differentiation, anti-FABP4 immunofluorescence staining revealed that ADSCs that differentiated into adipocytes appeared as accumulated lipid droplets in the cytosol (Figures 1(c) and 1(d)). Antiosteopontin immunofluorescence staining revealed that ADSCs that differentiated into osteocytes appeared as accumulated granules in the cytosol (Figures 1(e) and 1(f)). Finally, anti-collagen II immunofluorescence staining revealed that ADSCs that differentiated into chondrocytes appeared as a fibrous formation in the cytosol (Figures 1(g) and 1(h)).

### 3.2. Phosphorylation of Proteins

**Figure 2:**
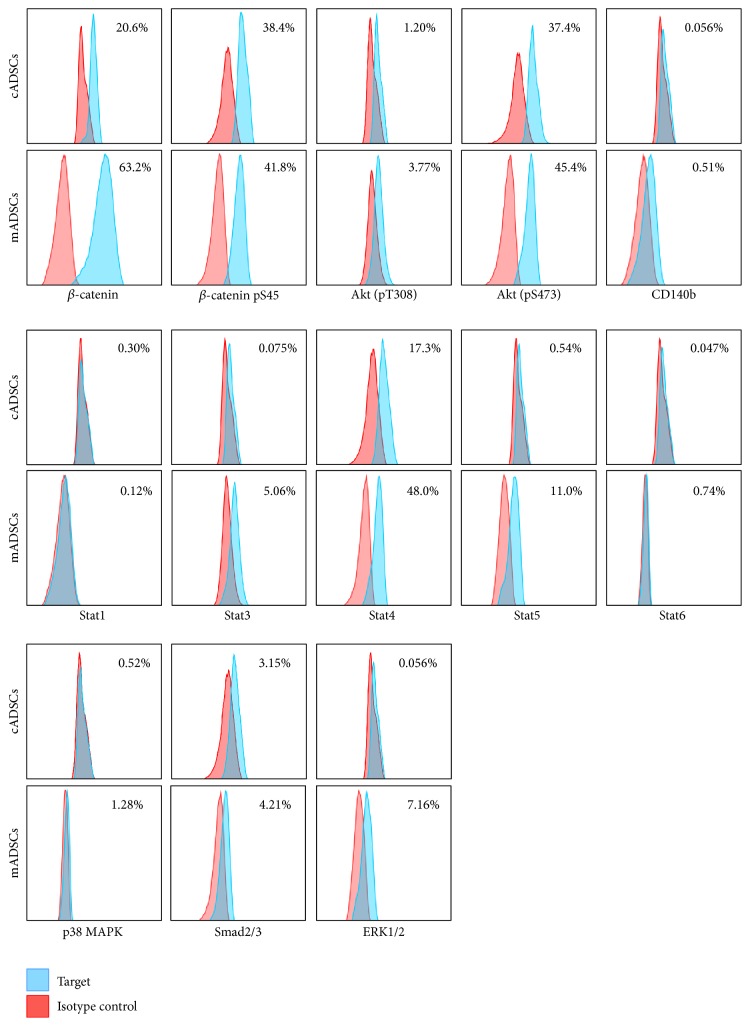
Comparison of protein phosphorylation of mADSCs and cADSCs. The red peaks represent the isotype controls, and the blue peaks represent antigens. *β*-catenin, *β*-catenin (pS45), Akt (pS473), Stat3, Stat4, Stat5, and ERK1/2 were detected, whereas Akt (pT308), CD140b, Stat1, Stat6, p38 MAPK, and Smad2/3 were not detected in mADSCs. In addition, *β*-catenin, *β*-catenin (pS45), Akt (pS473), and Stat4 were detected, whereas Akt (pT308), CD140b, Stat1, Stat3, Stat5, Stat6, p38 MAPK, Smad2/3, and ERK1/2 were negative in cADSCs. The percentages shown in the figure represent the positive rates of the antigen.

To clarify protein phosphorylation, *β*-catenin, *β*-catenin (pS45), Akt (pT308), Akt (pS473), CD140b, Stat1, Stat3, Stat4, Stat5, Stat6, p38 MAPK, Smad2/3, and ERK1/2 were measured using their respective antibodies. In mADSCs, *β*-catenin, *β*-catenin (pS45), Akt (pS473), Stat4, and Stat5 were detected, whereas Akt (pT308), CD140b, Stat1, Stat3, Stat6, p38 MAPK, Smad2/3, and ERK1/2 were not. Meanwhile, in cADSCs, *β*-catenin, *β*-catenin (pS45), Akt (pS473), and Stat4 were detected, and Akt (pT308), CD140b, Stat1, Stat3, Stat5, Stat6, p38 MAPK, Smad2/3, and ERK1/2 were not detected (Figure 2).

## 4. Discussion

In this study, we revealed that mADSCs and cADSCs have multipotency to differentiate into adipocytes, osteocytes, and chondrocytes. Both undifferentiated mADSCs and cADSCs expressed *β*-catenin, *β*-catenin pS45, Akt (pS473), and Stat4 phosphorylation and only mADSCs expressed Stat5 phosphorylation.

Previous studies revealed that WNT/*β*-catenin signaling inhibits adipogenesis in humans and mice. For example, the overexpression of Wnt1 or glycogen synthase kinase 3-b (GSK3b) phosphorylation-defective *β*-catenin mutant was shown to inhibit adipogenesis in 3T3-L1 preadipocytes (mouse fetal fibroblasts, which are widely used for metabolic study) by canonical pathway activation [7]. Another report revealed that pharmacological GSK3b inhibitors suppressed adipocyte differentiation [8].

However, to the best of our knowledge, there have been no previous reports on WNT/*β*-catenin signaling and ADSC adipogenesis in canines. In this study, we also revealed *β*-catenin and *β*-catenin pS45 phosphorylation in mADSCs and cADSCs. This suggests that cADSCs also inhibit adipogenesis with WNT/*β*-catenin signaling.

Akt, also known as protein kinase B, is a serine/threonine-specific protein kinase that plays roles in glucose metabolism, apoptosis, cell proliferation, transcription, and cell migration, among others [9]. In this study, we detected Akt pS473 phosphorylation in both mADSCs and cADSCs. Previous studies reported that Akt pS473 is phosphorylated by the mTOR-rictor pathway [10, 11]. We identified a previous report on Akt expression in cADSCs; however, it did not describe the phosphorylation site of Akt [12]. The relationship between ADSCs and Akt has been reported in mADSCs. A previous study showed the accumulation of intracytoplasmic lipids with Akt phosphorylation in ADSCs with the inhibition of ErbB2 activity [13]. In murine 3T3-L1 preadipocytes, the inhibition of adipocyte differentiation was also found when Akt was inhibited [14]. Akt phosphorylation is also necessary for the differentiation of brown adipocytes, and PI3K inhibition blocks adipocyte differentiation in mice [15].

In this study, we detected the phosphorylation of Stat4 and Stat5 in mADSCs, but only that of Stat4 in cADSCs. A previous study revealed Stat4 expression at the mRNA level in mADSCs [16]. However, we found no reports on Stat4 expression at the protein level. Therefore, we concluded that this is the first report on Stat4 expression in mADSCs and cADSCs.

Many clinical trials have been conducted for the ADSC-related treatment of cardiovascular disease, spinal cord injury, cirrhosis, renal insufficiency, and skin fistula with Crohn's disease and for breast reconstruction after mastectomy in humans [17]. In veterinary medicine, we can also find some clinical trials for the ADSC-related treatment of acute thoracolumbar intervertebral disc disease [18], inflammatory bowel disease [19], chronic osteoarthritis [20], and so on. However, there has been insufficient basic research on cADSCs in veterinary medicine and the clinical trials that have been conducted provided limited evidence. In previous studies, comprehensive information on surface antigen markers or gene expression was reported for human ADSCs and mADSCs [5, 21, 22]. However, no comprehensive study on phosphorylation has been performed for a long time. In this study, some phosphorylated proteins were found to be expressed in undifferentiated mADSCs and cADSCs. However, it has not been revealed which signal pathways are expressed to maintain stemness in undifferentiated mADSCs and cADSCs. Therefore, it is necessary to investigate which signal is important to maintain stemness. Nonetheless, the present study provides a useful foundation for future research in veterinary medical science.

## 5. Conclusion

mADSCs and cADSCs showed adipogenic, osteogenic, and chondrogenic differentiation potential. mADSCs and cADSCs induced similar phosphorylation of *β*-catenin and Akt, in contrast to that of the Stat family.

## Figures and Tables

**Table 1:** Antibodies used in this study.

Target molecule	Abbreviation	Species isotype	Label	Company	Clone	Dilution
Phosphorylated Human *β*-Catenin (pS45) Peptide	*β*-catenin (pS45)	Mouse (BALB/c) IgG1, *κ*	Alexa Fluor 647	BD Biosciences	K63-363	1 : 50
Mouse *β*-Catenin aa. 571-781	*β*-catenin	Mouse IgG1	Alexa Fluor 488	BD Biosciences	14/Beta-Catenin	1 : 200
Phosphorylated Human Akt1 (pT308) Peptide	Akt (pT308)	Mouse IgG1, *κ*	Phycoerythrin	BD Biosciences	J1-223.371	1 : 50
Phosphorylated Human Akt1 (pS473) Peptide	Akt (pS473)	Mouse (BALB/c) IgG1, *κ*	Alexa Fluor 647	BD Biosciences	M89-61	1 : 50
Phosphorylated Human PDGFR*β* (pY1009) Peptide	CD140b	Mouse IgG2b, *κ*	Phycoerythrin	BD Biosciences	J25-602	1 : 50
Phosphorylated Human Stat1 (pY701) Peptide	Stat1	Mouse IgG2a	Alexa Fluor 488	BD Biosciences	4a	1 : 50
Phosphorylated Human Stat3 (pY705) Peptide	Stat3	Mouse IgG2a, *κ*	Phycoerythrin	BD Biosciences	4/P-STAT3	1 : 50
Phosphorylated Human Stat4 (pY693) Peptide	Stat4	Mouse IgG2b, *κ*	Alexa Fluor 647	BD Biosciences	38/p-Stat4	1 : 50
Phosphorylated Human Stat5 (pY694) Peptide	Stat5	Mouse IgG1, *κ*	Alexa Fluor 488	BD Biosciences	47/Stat5 (pY694)	1 : 50
Phosphorylated Human Stat6 (pY641) Peptide	Stat6	Mouse IgG1, *κ*	Phycoerythrin	BD Biosciences	J71-773.58.11	1 : 50
Phosphorylated Human p38 MAPK (pT180/pY182) Peptide	p38 MAPK	Mouse IgG1, *κ*	Phycoerythrin	BD Biosciences	36/p38 (pT180/pY182)	1 : 50
Phosphorylated Human Smad2 (pS465/pS467)/Smad3 (pS423/pS425) Peptide	Smad2/3	Mouse IgG1, *κ*	Alexa Fluor 647	BD Biosciences	O72-670	1 : 200
Phosphorylated Rat ERK1 (pT202/pY204) Peptide	ERK1/2	Mouse IgG1	Alexa Fluor 488	BD Biosciences	20A	1 : 50
